# Noncontact Detection of Respiration Rate Based on Forward Scatter Radar

**DOI:** 10.3390/s19214778

**Published:** 2019-11-03

**Authors:** Fan Yang, Zhiming He, Yuanhua Fu, Liang Li, Kui Jiang, Fangyan Xie

**Affiliations:** 1 School of Information and Communication Engineering, University of Electronic Science and Technology of China, Chengdu 611731, China; zmhe@uestc.edu.cn (Z.H.); f_yuanhua@163.com (Y.F.); riemail@163.com (L.L.); 18847149074@163.com (K.J.); xiefangyan@uestc.edu.cn (F.X.); 2 College of Physics and Electronic Information, Inner Mongolia Normal University, Hohhot 010022, China

**Keywords:** forward scatter radar, noncontact detection, RCS, respiration rate

## Abstract

Bioradar-based noncontact breathing detection technology has been widely studied due to its superior detection performance. In this paper, a breath detection mechanism based on the change in radar cross section (RCS) is proposed by using a forward scatter radar and the deduction of the mathematical model of the received signal. Furthermore, we completed human breathing detection experiments in an anechoic chamber and in an ordinary chamber; we obtained the breathing rate through envelope detection in cases where the human orientation angle was 0, 30, 60, and 90°. The analysis of the measured data shows that the theoretical model fits well with the measured results. Compared with the existing single-base radar detection schemes, the proposed scheme can detect human respiratory rates in different orientations.

## 1. Introduction

Respiratory rate is an important index reflecting human physiological states. Firstly, whether a life exists or not can be determined by the respiratory state. Secondly, parametric abnormalities of respiratory activities often reflect health emergencies such as respiratory choking and asthma. Finally, humans in cars, indoor spaces, and other special environments can be effectively protected through long-term intelligent monitoring of human respiratory states. Microwave-based noncontact detection is irreplaceably advantageous in convenience in comparison with contact respiration detection. Therefore, electromagnetic-wave-based noncontact respiration detection has recently become a hot research topic. Bioradars are widely used in medical applications such as sleep monitoring [1] and SIDS detection [2]. In general, noncontact respiration detection is carried out using the following two approaches.

Ultra-wideband (UWB) radar [3,4,5,6]: The UWB radar works by transmitting periodical pulse signals at the transmitter. The pulse period is longer than the transmission path duration, so near-field clutter is separated from echoes which contain target information in the time domain. Hence, near-field clutter and power leakage of the transmission can be mitigated with the UWB radar. In addition, low power consumption can be easily designed for the UWB radar due to its large bandwidth and low average power consumption. Nevertheless, the application of UWB radar is generally limited due to the high cost of the hardware.

Continuous wave (CW) radar: Compared with UWB, the CW radar has a simpler structure. Several types of structures are generally applied in CW radar, such as the homodyne structure [7], double-sideband structure [8], and direct intermediate frequency sampling structure [9].

Unlike UWB radars, the propagation delay time cannot be measured with CW radars, making it difficult to obtain distance information. In addition, the transmitter is coupled with the receiver in a CW radar. Hence, direct current bias and low-frequency noise are generated, which influence detection results. Nevertheless, the simple hardware structure and low hardware cost of CW radars are always needed in the industry. In this regard, Sandra Costanzo proposed low-cost flexible respiration detection with a universal software radio platform (USRP) [10]. In addition, Carolina Gouveia summarized a solution to random movement in aspects of hardware and signal processing [11].

Taking the above discussions into consideration, few works examine breath detection with random orientation of the test subject. The radars based on the biological mechanism of chest wall micromotion do not perform well enough without the correct orientation of the antennas and of the test subject. During sleep monitoring, the test subject cannot be asked to maintain one posture throughout the night. When the test subject sleeps on their side, the antenna cannot be facing towards their chest wall. As a result, effective information can hardly be detected with radars based on chest wall micromotion detection principles. In addition, during monitoring of a driver’s respiration, the detection error of a single-static radar will be increased when the driver twists their body while operating the steering wheel.

The problems mentioned above must be solved. Specifically, radars should be insensitive to human body rotation, and their hardware structures need to be simplified in their realization. Meanwhile, the hardware cost must be brought down, and noise generated from random human motion must be removed. In this way, human respiratory rate can be accurately detected with bioradars. To solve these problems, we propose a respiratory rate detection scheme by exploiting forward-scatter-based bioradar. In this system, the transmitter and receiver are arranged on opposite sides of a person so that their abdomen is placed on the baseline. The radar cross-section (RCS) will change during human respiration, causing a change in the received signal amplitude. The respiration curve can be obtained through envelope detection of the received signal.

The main contributions of this paper can be summarized in the following:

1. Considering the disadvantage of traditional detection in that it relies on chest wall micromovement but is sensitive to random orientation of the human, a novel detection scheme based on human RCS changes is proposed. Respiration-based changes of RCS are analyzed. Then, the analytical expression of the received signals is derived.
2. To confirm the theoretical model, we conducted the respiration detection experiments in an anechoic chamber with a forward scatter radar. Experimental results show that the respiratory rate is accurately detected by the proposed scheme.
3. To test the system’s performance in different orientations, the FSR system for calibration of the contact sensor was completed indoors. The verification results show that the system is insensitive to human rotation. The respiratory rate can still be measured with rotation of less than 90°. The reliability of the theoretical model is verified by the experimental data.

## 2. Theoretical Model

During respiration, the chest volume is changed due to muscle contraction and relaxation. As a result, a pressure difference is generated between the internal and external environments of the chest. Air flows from the high-pressure area to the low-pressure area, periodically entering and exiting the lungs. The motion of the thorax and abdomen is driven by respiratory motion. The body shape is changed as a result of abdominal motion. Consequently, the RCS is changed, and thus, the amplitude of the received signal changes. In this way, the human respiratory state is detected. A radar system based on this biological mechanism, forward scatter radar (FSR)—which is sensitive to RCS changes—is introduced.

**Figure 1:**
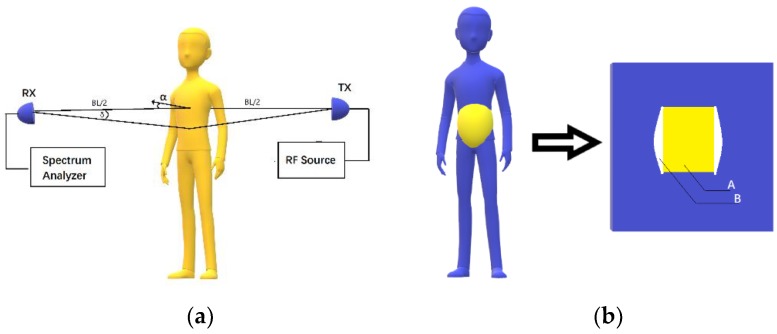
(**a**) Forward scatter radar structure; (**b**) changes in the frontal projection of the human abdomen during breathing.

As a special bistatic radar system, FSR is characterized by a bistatic angle reaching up to 180°. Existing studies mainly focus on FSR detection of objects such as stealth aircraft, unmanned aerial vehicle (UAV), and automobiles [12,13]. FSR has a very interesting feature: With a bistatic angle of less than 180°, the change of RCS only depends on the projection outline area of an electrical conductor and the wavelengths of electromagnetic wave signals; the surface wave absorption features of the target are irrelevant (it is insensitive to clothing textures during human respiration detection). To the best of our knowledge, respiration detection with FSR has not been well investigated in existing literature. The main idea of most existing studies is to detect the micro-Doppler features changed by chest wall micromotion. In this paper, we found that the amplitude of the received signal changes with the changes as a result of human respiration. Figure 1 shows the RCS change pattern of the FSR radar.

As shown in Figure 1a, BL is the baseline, namely, the connecting line between the transmitter and the receiver. α is the included angle between the normally-oriented human and the baseline; it is defined as the orientation angle. δ is the antenna beam angle.

In this paper, the scattered field generated by human surface currents and the electromagnetic field of human penetration by electromagnetic waves are neglected. Therefore, we have the following equation:

(1) $$E_{{}^{\mathrm{re}}}=E_{0}-E_{{}^{\mathrm{sh}}}$$

*E*_re_ denotes the electric field from the signal to the receiver and *E*_0_ denotes the electric field without the body. The power of the transmitter is constant, so *E*_0_ does not change. *E*_sh_ is a shadow field generated by the human body and is linearly correlated with RCS generated within the scope of antenna exposure on the human body. *E*_sh_ varies with the change of human RCS. Computation of near-field RCS is very complicated. However, RCS of the target—denoted by *σ*_b_*(**r**)*— can be obtained using Equation (2) when the bistatic angle approaches *β_b_* = 180°, according to the RCS computation equation proposed by Chernyak [14]:

(2) $$\sigma_{b}(r)=\frac{4\pi }{\lambda^{2}}{\left|\int_{A_{t}}\exp[j(\frac{2\pi }{\lambda })\rho r]dS\right|}^{2}$$

In Equation (2), *A_t_* is obtained through Babinet’s principle and refers to the aperture of the accurate human outline within the scope of antenna exposure. ***ρ*** denotes the radial component at a random point in the effective aperture, ***r*** denotes the unit vector in the direction of the receiver, and *λ* denotes the wavelength of the transmitted signal.

As shown in Equation (2), ***ρ*** is perpendicular to ***r*** under *β_b_ =* 180°. Then, Equation (3) is obtained.

(3) $$\sigma_{b}(180{}^{\circ})=4\pi (\frac{S_{t}}{\lambda }{)}^{2}$$

where *S_t_* refers to the area of the equivalent aperture. According to [15,16], the theory is accurate when *β_b_ =* 180*°* and *BL > 2D^2^/λ*, where *BL* denotes the distance between a human body and the transmitter or the receiver and D denotes the maximum antenna size. Furthermore, the following equation is obtained using Equation (3):

(4) $$\sigma_{b}(180^{\circ })=G_{t}S_{t},G_{t}=4\pi \frac{S_{t}}{\lambda^{2}}$$

where G_t_ denotes the antenna’s gain of uniform exposure with the area of *S_t_*. The change of RCS in human respiration can be analyzed according to Equation (4).

**Figure 2:**
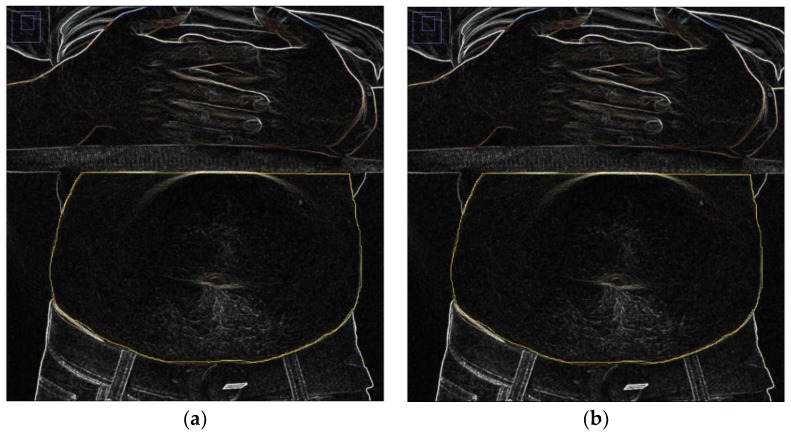
Use of imageJ to measure the projected frontal area on exhalation and inhalation (**a**) inhalation status; (**b**) exhalation status.

As seen in Figure 1a, the thorax expands and the abdomen gets larger during human inhalation, so RCS increases. During exhalation, the external intercostal muscles and diaphragm muscles relax, and the abdomen gets smaller, so RCS decreases. As seen in Figure 1b, RCS values can be estimated with the Babinet method. Using a 34-year-old adult male as an example, the abdomen width is 25 cm during inhalation and 29 cm during exhalation within the antenna exposure area. Furthermore, the human projection can be roughly interpreted as a rectangle that is 25 cm wide and 25 cm high (inhalation state). During exhalation, the waist gets larger, and Figure 1b is added onto the projection face. As seen in the quantitative analysis results in Figure 2a,b, the human abdomen was observed at the orientation angle *α* = 0° during exhalation and inhalation. We placed another black belt at 26 cm above the waistband. Then, after the detection of edges with ImageJ, the change in the abdomen area was obtained through the processing of these pictures. Finally, the rate of expiratory area to inspiratory area is equal to 95.483%. According to Equation (3), the RCS has decreased to 91.17%. *ξ* is the RCS attenuation coefficient to quantify the change of the RCS.

Forward scatter radar (FSR) was designed for respiration rate detection according to the above analysis. In comparison with traditional detection techniques, FSR has advantages in analysis of RCS changes during human respiration and is insensitive to human rotation. It can be observed in Figure 1 that RCS at the exhalation and inhalation states changes as a whole after human rotation. 

In the FSR, a single-frequency continuous wave signal is sent by the transmitter. The transmitted signal *T*(*x*) can be expressed as:

(5) T(x)=Acos(ωt+φ)

where *A* is the amplitude and *φ* is the initial phase. During human respiration, the thoracoabdominal motion changes with respiration, thus leading to the change in RCS. As a direct consequence, the amplitude of the received signal is changed and *A* turns into *A(t)* varying with time, or *t*. Let *ξ* denote the RCS ratio between exhalation RCS and inhalation RCS, where 0 < *ξ* < 1.

(6) $$\xi =\frac{RCS_{exhale}}{RCS_{inhale}}=\frac{E_{exhale}}{E_{inhale}}$$

*B(t)* ∈ [−1,1] is deemed as a normalization function for the change of the respiratory capacity. We assume that RCS is linearly influenced by human respiratory motion. The following equation can be obtained:

(7) $$E_{sh}(t)=\frac{E_{inhale}+E_{\mathrm{ex}hale}}{2}-\frac{E_{inhale}-E_{\mathrm{ex}hale}}{2}B(t)$$

where *E_sh_(t)* denotes the electric field strength of the shadow field, *E_inhale_* is the electric field strength of the shadow field during inhalation, and *E_exhale_* denotes the electric field strength of the shadow field during exhalation. According to Equation (1), we have:

(8) $$A(t)=E_{0}-\frac{E_{inhale}+E_{\mathrm{ex}hale}}{2}+\frac{E_{inhale}-E_{\mathrm{ex}hale}}{2}B(t)$$

where *E*_0_ denotes the amplitude of the received signal for the case that there is nobody in the field. Incorporating Equation (6) into Equation (8), we get:

(9) $$A(t)=E_{0}-(\frac{1+\xi }{2})E_{\mathrm{inhale}}+\frac{1-\xi }{2}E_{\mathrm{inhale}}B(t)$$

According to Equation (9), the change in amplitude can be deemed as a course of amplitude modulation. During exhalation, amplitude of the received signal is at its maximum, i.e., *A_MAX_ = E*_0_ – *ξE_inhale_*, while during inhalation, the amplitude is at its minimum, namely *A_MIN_ = E*_0_ – *E_inhale_*. In a practical application, *E*_0_, *A_MAX_,* and *A_MIN_* can be measured easily.

## 3. Experiments and Analysis

### 3.1. Experiment 1: Respiration Detection Testing in a Anechoic Chamber

In this subsection, we present the experiment results obtained in an anechoic chamber of the University of Electronic Science and Technology. In an anechoic chamber, external radio interference can be effectively absorbed. Hence, the influence of electromagnetic radio waves of the surroundings on the reflected signals is negligible. This test scenario is suitable for the theoretical received signal model. This experiment aims to analyze the characteristics and amplitudes of the received signal in the time domain during detection of human respiration using the FSR system.

Experimental steps: A single-frequency continuous wave signal of 2.4 GHz was generated by the radio source TSG4102. The emitted signal amplitude was −30 dBm. The receiving antenna was directly connected to the portable spectrum analyzer RSA306B produced by Tektronix. The frequency of RSA306B ranged 9 kHz–6.2 GHz. The measurement scope was from +20 dBm to approximately −160 dBm. The experimental requirements were satisfied.

**Figure 3:**
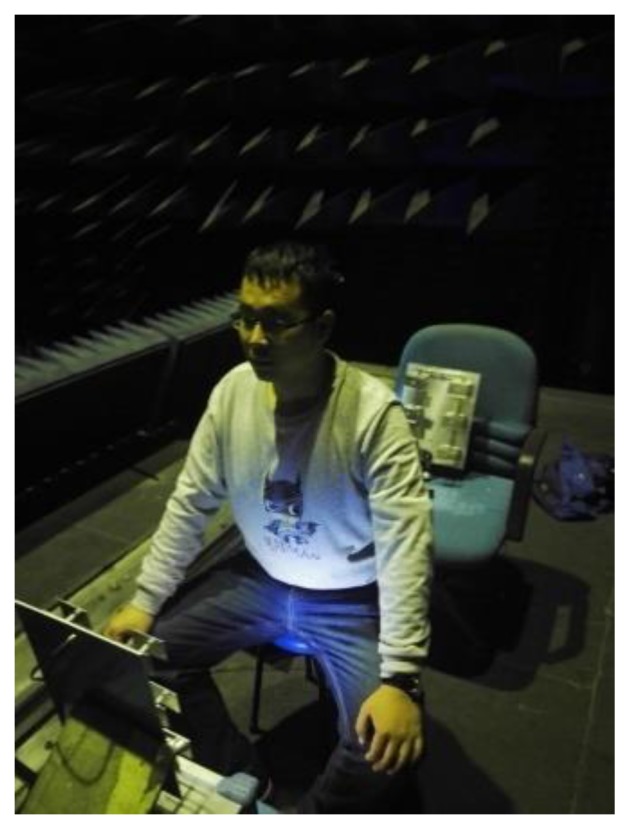
Experiment setup in the anechoic chamber.

The time domain of the signal could be directly obtained with RSA306B. As is shown in Figure 3, we used a copper sheet antenna, which was oriented towards the subject’s back; the antenna’s parameters and radiation patterns can be found in [17]. The horizontal wave lobe was 30°, and the vertical wave lobe was 20°. The subject stayed at the midpoint between the transmitter antenna and the receiver antenna. The transmitter antenna, the subject, and the receiver antenna were located on the same straight line. The distance between the transmitter and the receiver was 1.6 m. The signal time scope was 14 s. The distance between the transmitter and the subject was 71 cm. Five respiration cycles were completed by the subject.

**Figure 4:**
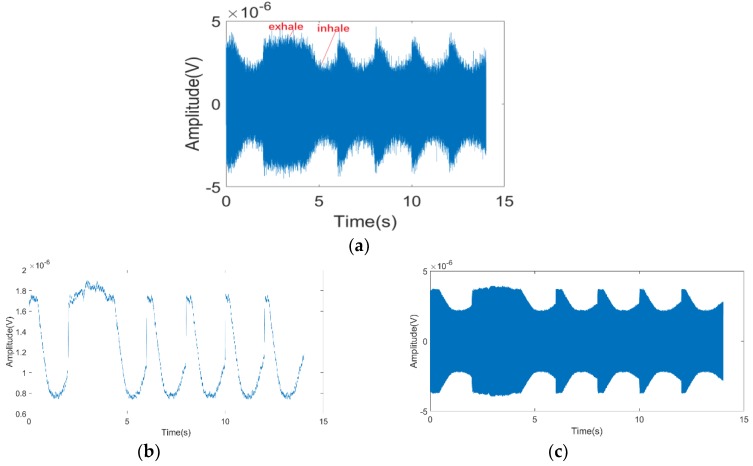
(**a**) The received signal of the forward scatter radar (FSR) system in the anechoic chamber; (**b**) the output of the envelope detection; (**c**) the simulated signal for the analytical model.

According to the experimental results shown in Figure 4a, the maximum signal amplitude was 4.7 μV, and the minimum signal amplitude was 2.5 μV. It was shown that the power of signals received through FSR was very low, but the signal was still clearly shown by its amplitude modulation and with its obvious envelope. The same voltage-controlled oscillator could not be used for the bistatic radar; during analysis of the frequency bias, the transmitter would be influenced by temperature drift, etc. Hence, during the analysis of frequency changes, the influence of temperature drift or respiration on the frequency changes could not be distinguished. However, the system was verified to detect the respiration rate through analysis of only the amplitude.

Furthermore, envelope detection of the signal was carried out. A respiration curve was directly obtained. As shown in Figure 4b, at each peak, the subject clearly shook during the five complete courses of respiration. This shaking was deemed to be generated by micromotion on the skin’s surface during respiration.

Finally, in accordance with the theory mentioned above, we normalized the respiratory curve obtained in Figure 4b to [−1,1] and carried out the simulation to obtain Figure 4c based on Equation (9). By comparing Figure 4a,c, we found that although we lost the information caused by skin micromovement, we can still acquire the respiration rate from the envelope of the waveform.

### 3.2. Experiment 2: Respiration Detection in a Common Room

**Figure 5:**
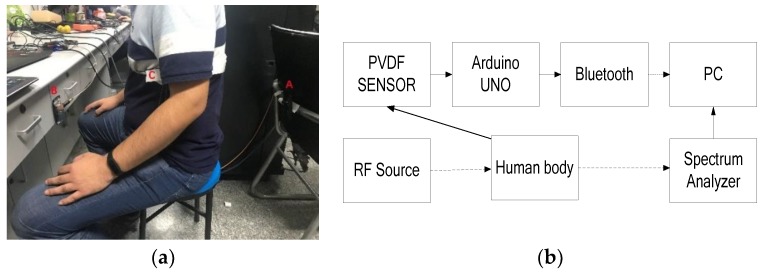
(**a**) Experiment setup in the common room; (**b**) the experiment system block diagram.

**Figure 6:**
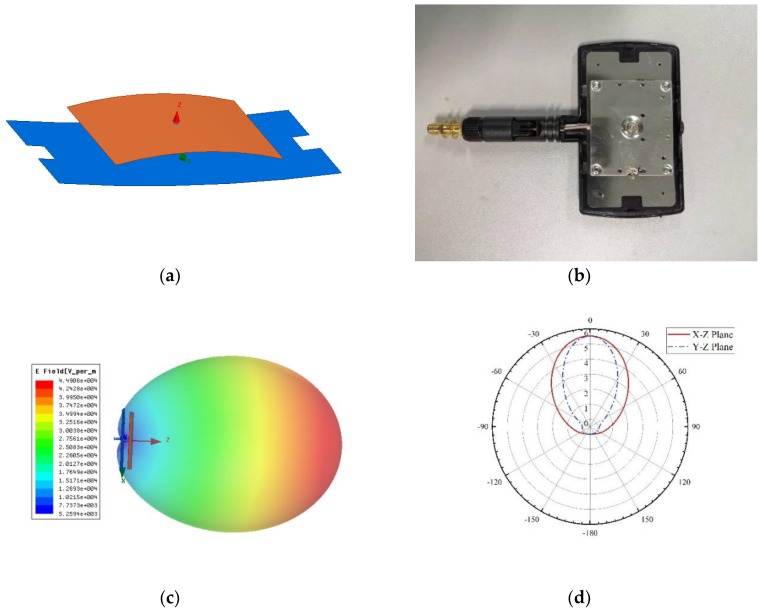
The antenna’s information (**a**) the antenna model; (**b**) antenna prototype; (**c**) radiation pattern in 3D; (**d**) radiation pattern.

For indoor testing, the experimental results of the proposed system were compared with a contact-sensor-based detection system. In this experiment, a PVDF (polyvinylidene fluoride) piezoelectric film sensor (IPS-17020, manufactured by ZHIMK company in Shenzhen, China) was used. The respiratory rate could be accurately detected with the contact detection system. Meanwhile, a universal WIFI directional antenna with a lower cost was used instead of a copper sheet antenna. Figure 5 shows the diagram of the experiment. In Figure 5a, A is the transmitter antenna, B is the receiver antenna, and C is the PVDF sensor, which was positioned around the chest. The corresponding details of the antenna are presented in Figure 6. The horizontal wave lobe was 105°, and the vertical wave lobe was 70°. As seen in the experiment shown in Figure 5a, the distance between the transmitter and the receiver was 76 cm. The subject turned his back to the transmitter, while sitting in the midpoint between the transmitter and receiver. The distance between the subject’s back and the transmitter was 20 cm. The height of the antennas and of the navel was 65 cm.

**Figure 7:**
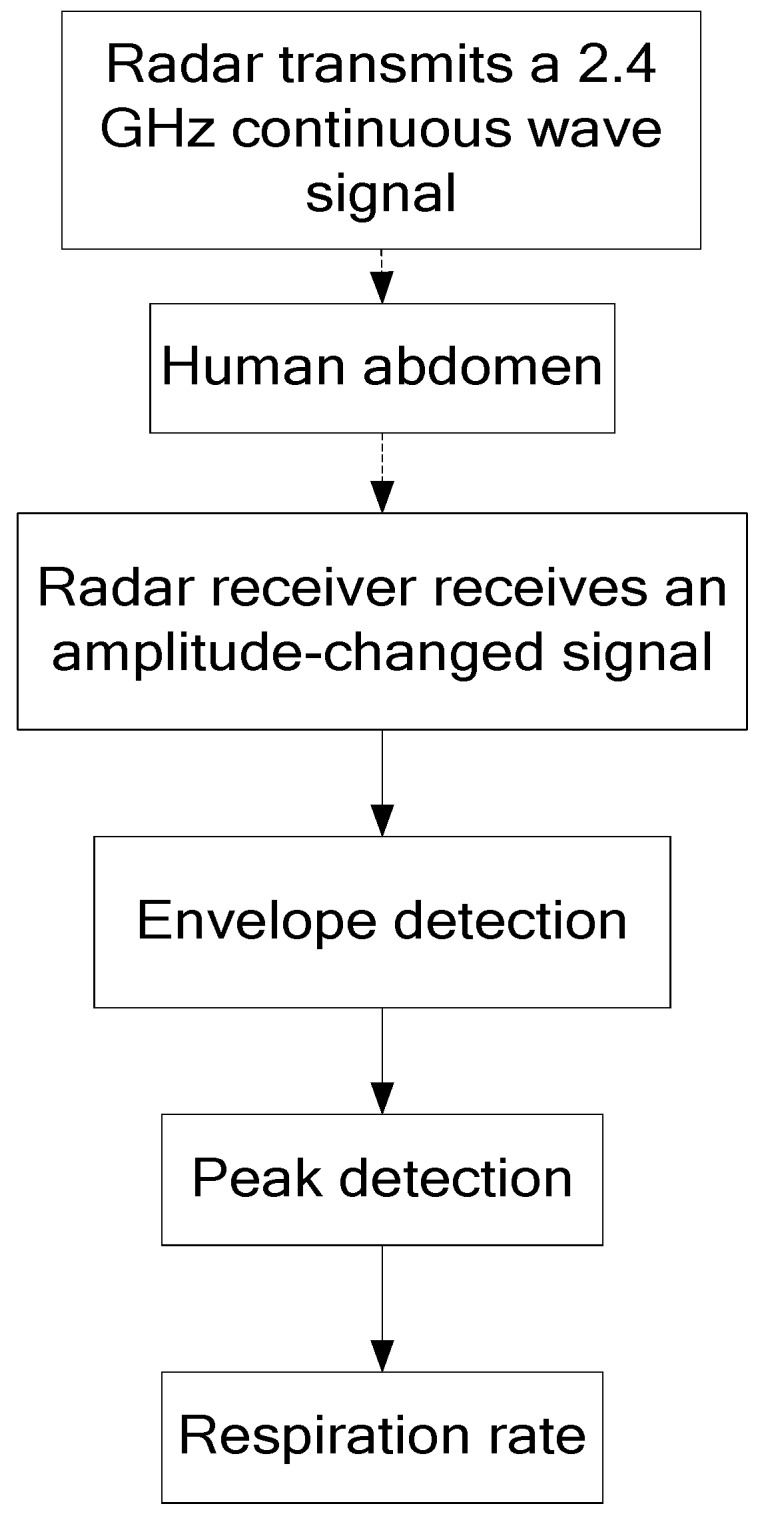
The flowchart of the proposed FSR system.

**Figure 8:**
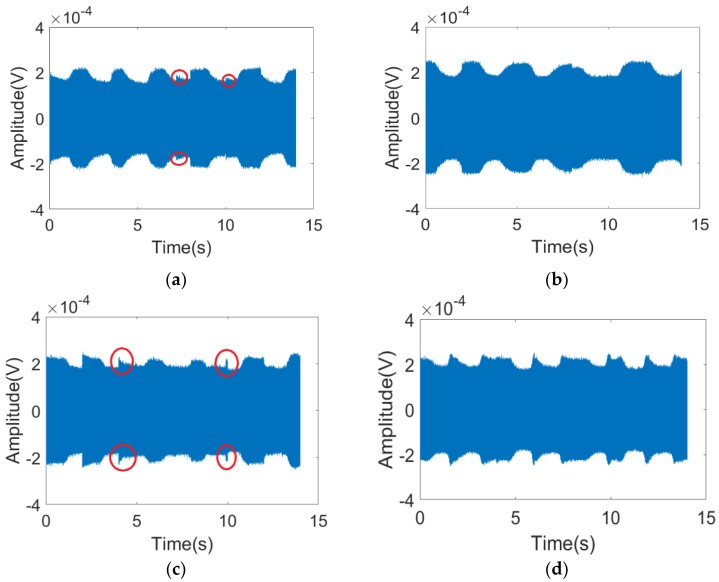

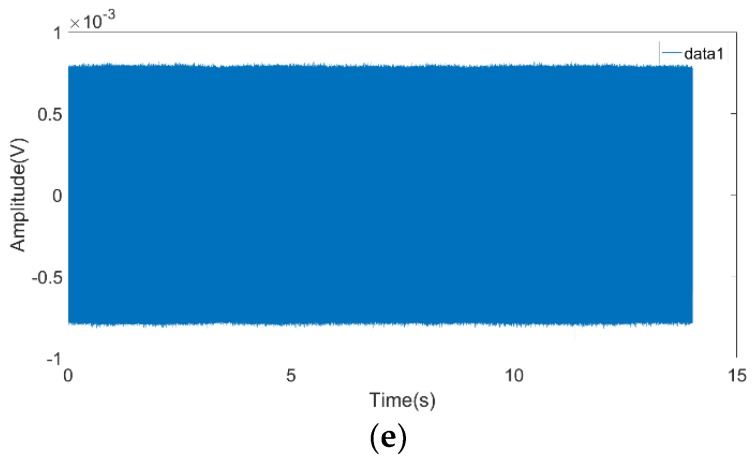
The received signal of the FSR system with a different orientation angle α for (**a**) 0°; (**b**) 30°; (**c**) 60°; (**d**) 90°; (**e**) the received signal of the FSR system with nobody in the test area.

The whole experimental process is displayed in Figure 7. First, the radar emitted a 2.4 GHz continuous wave signal. When the radio wave had a diffraction around the abdomen, the change in belly size modulated the carrier in amplitude. Second, at the radar receiver, the breath curve was acquired through envelope detection. Finally, the respiration rate could be perceived after peak detection. The respiration detection results of FSR were measured at orientation angles α of 0, 30, 60, and 90°, respectively. The experiment results are shown in Figure 8.

**Figure 9:**
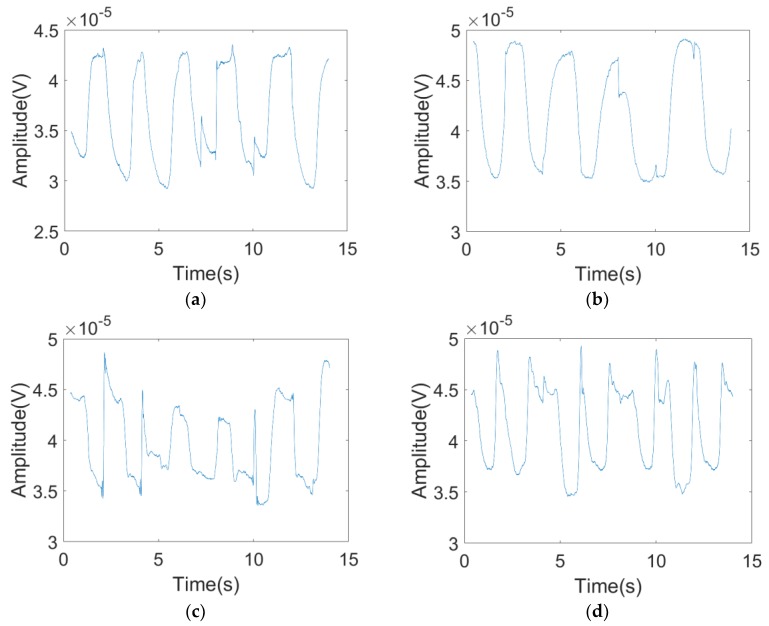
The output of the envelope detection with the different orientation angle α for (**a**) 0°; (**b**) 30°; (**c**) 60°; and (**d**) 90°.

It can be observed in Figure 8 that the detection results of respiration rates were good under the orientation angles of 0, 30, 60, and 90°. In the diagram, peaks marked with round circles were generated from random physical motion. It was found that human motion was very short in the time line in comparison with respiration; it could be easily distinguished from respiration through observation within a continuous time threshold. The output of the envelope detection is shown in Figure 9.

**Figure 10:**
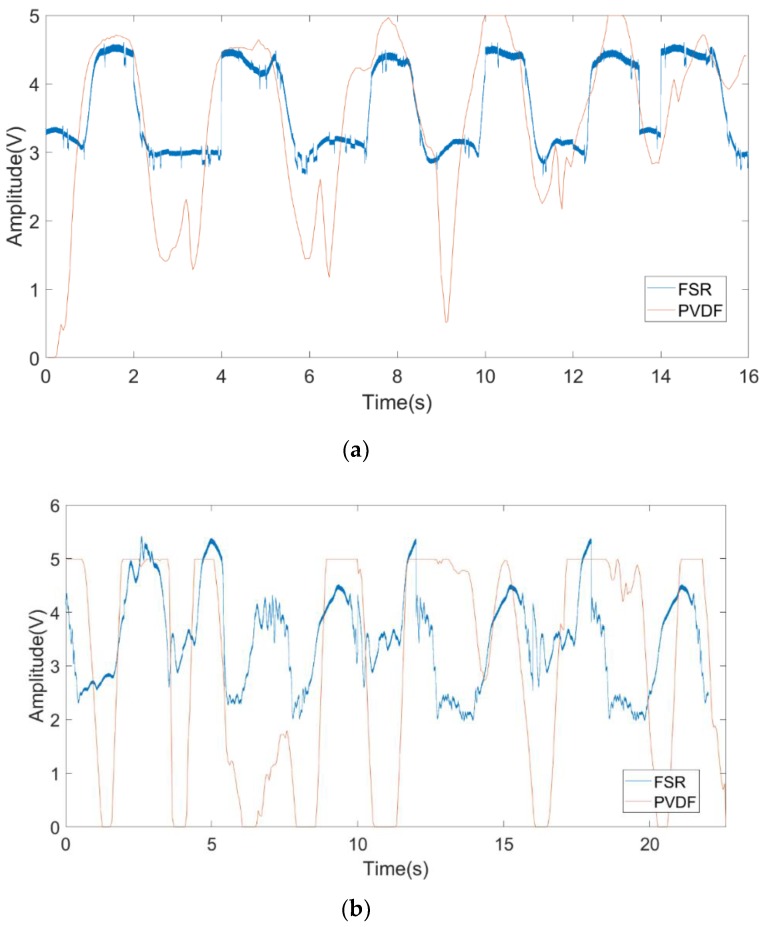
The comparison between FSR and PVDF (polyvinylidene fluoride) with the different orientation angle α for (**a**) 0°; (**b**) 90°.

Finally, to demonstrate the effectiveness of the proposed FSR system, we compared PVDF with FSR signals in the cases where the angle was 0 or 90°. We executed the experiment with the same setup used in the second experiment. The results of multiplying the FSR signal with a scaling factor are shown in Figure 10. In Figure 10a, it can be observed that the FSR signal fits well with the PVDF signal when the angle is 0°; the respiration rate is 22.5 times per minute. The respiration rate obtained from the PVDF signal is the same as that from the FSR signal. In Figure 10b, when the angle is 90°, the two curves are slightly different due to the fact that the waveform of the FSR changes more violently when the orientation angle is 90°. RCS is influenced by the changing of orientation angles. There was more fat on the front face of abdomen, so skin micromotion was more violent during its expansion and contraction. In Figure 10b, although there are mismatches between the PVDF signal and the FSR signal, both the PVDF signal and the FSR signal show the same respiration rate. At the angle of 90°, the respiration rate is 19.2 times per minute.

The quantitative analysis results were as follows: The signal received by the system satisfied *E*_0_ = 800 μV, as measured in Figure 8e according to Equations (5) and (6) when there was nobody in the field. Based on diagrams A, B, C, and D, the maximum amplitude *A_MAX_* and minimum amplitude *A_MIN_* during respiration were listed in Table 1.

*ξ_c_* is calculated using Equations (10) and (11). For example, using the angle of 0°, the result of the analysis of *ξ* in the theoretical model is 91.17% (mentioned in Section 2); 3.83% error can be observed mainly due to the fact that the human body is not a perfect conductor, so part of the signal cannot go through the body.

(10) $$\{\begin{array}{l} A_{MAX}=E_{0}-\xi E_{inhale}\\ A_{MIN}=E_{0}-E_{inhale}{}_{} \end{array}$$

(11) $$\xi_{c}=\frac{E_{0}-A_{MAX}}{E_{0}-A_{MIN}}$$

Finally, the results of Experiments 1 and 2 were compared. It was found that the system had a high robustness with respect to the subject’s clothing. The first experiment was made in winter. The subject wore two layers of clothes. The first layer was made of chemical fibers and the second layer was made of cotton. The second experiment was made in summer, and the subject wore one layer of clothes made of cotton. Obviously, despite the texture, thickness, and number of layers of clothes, the system’s detection results were good due to the characteristics of FSR.

## 4. Conclusions

This paper proposes a human respiratory rate detection method based on FSR. In this method, respiratory rates were obtained via the envelope detection of received signals according to the changes in RCS caused by peoples’ breathing. Compared with traditional methods, it has the advantages of easy realization and lower computational complexity. Moreover, the analytical expression of the received signal was derived and the system’s effectiveness in detecting human respiratory rate was analyzed under different angles. The experiment’s results show that the proposed approach is able to detect respiratory rates accurately under different measuring conditions. From the viewpoint of future development, the technique of detecting the respiratory rate via FSR has great practical value, as it can be applied to the realization of systems for sleep monitoring between cell phones and routers, as well as for the detection of drivers through the cooperation of cars and cell phones.

## Figures and Tables

**Table 1:** The experiment results at different angles.

*α*	*A_MAX_* (μV)	*A_MIN_* (μV)	*ξc*
0°	227	144	87.34%
30°	255	180	87.9%
60°	240	170	88.8%
90°	240	175	89.6%
